# Developing an index for measuring gender lens investing in organizations: the GLIMETRICS framework

**DOI:** 10.3389/fpsyg.2025.1534355

**Published:** 2025-04-30

**Authors:** Maite Palomo-Vadillo, Ana-Lucia Ortega-Larrea, María-Julia Bordonado-Bermejo, Carmen De-Pablos-Heredero

**Affiliations:** ^1^Business Administration Department, ESIC University, Madrid, Spain; ^2^Humanities Department, ESIC University, Madrid, Spain; ^3^Business Administration and Law Department, ESIC University, Madrid, Spain; ^4^Business Administration Department, Rey Juan Carlos University, Madrid, Spain

**Keywords:** gender lens investing (GLI), Delphi method, gender issues, value creation, gender metrics

## Abstract

**Introduction:**

The objective of this article is to develop and validate a metric for assessing gender lens investing (GLI) practices within organizations. The validation of these measurement items constitutes a methodological innovation, responding to calls in the literature for the exploration of novel approaches in comparative studies.

**Methods:**

To achieve this objective, the development of the measurement items was informed by a comprehensive literature review, and their content validation was conducted through a Delphi study.

**Results:**

The primary outcome of this research is the recommendation and validation of a tool designed to measure various dimensions of GLI practices, from both an academic standpoint and the perspective of expert evaluators.

**Implications:**

The application of this tool is expected to facilitate the integration and generalization of diverse perspectives on GLI. As Investment Fund Agencies and companies increasingly invest in GLI initiatives, there is a growing demand for robust instruments to effectively assess their impact.

## 1 Introduction

Gender lens investing (GLI) practices refer to a movement that, for ethical reasons, establishes or capitalizes funds and companies supporting women as leaders and throughout society (Roberts, 2016). The idea that women should have access to training and jobs in all areas of work, receive equal pay, be able to reconcile work and family life on equal terms with men, and that companies should be organized not only by men but also by women who understand the diverse needs of consumers, aligns with key objectives in organizational psychology. According to Chmiel et al. (2017), in ‘organizational psychology’, psychology plays a central role, as it pertains not only to management but also to how managers lead, how coworkers interact, and how organizations function. In this sense, measuring the effectiveness of GLI policies in companies means assessing whether organizational psychology accounts for gender diversity.

Initial measurements focused on economic impact and risk distribution (Criterion Institute, 2020). Indeed, similar to environmental, social, and governance funds (ESG), investing with a gender lens has proven advantageous for companies. This approach not only fosters a sense of safety and inclusion among but also leads to better long-term economic results (Subramanian, 2022). In fact, numerous reports have demonstrated the importance of this concept, including “Just Good Investing: Why Gender Matters to your Portfolio and What You Can Do about it” (Calvert Impact Capital, 2019), “Gender Lens Investing Landscape. East & Southeast Asia” (2020), “Gender Lens Investing: Legal Perspectives” (2021), which discusses the incorporation of gender considerations in loans and in equity investment, and the “Gender Lens Investing Report” (2021), among others. All these reports reference projects funded by investment funds (equity or public), yet it is reasonable that the portfolios themselves are not publicly disclosed. Nonetheless, private investment in such funds is on the rise, and therefore, some companies are mentioned as business cases (Sasakawa Peace Foundation, Catalyst at Large, and SAGANA, 2020).

However, the initial initiatives to develop a tool for measuring investment with a gender lens seem not to have studied the earlier attempts at measurement. These initiatives either focus on different aspects to be measured, use different metrics, or address different fronts such as investment funds, small businesses, entrepreneurship, governance, etc. Among the most recent initiatives, Sustainable Finance Geneva suggested five infrastructures along with their respective tools for measuring GLI criteria (2021):

1. EDGE (Economic Dividends for Gender Equality). It assesses the gender balance within organizations across their talent pipeline, examines pay equity, evaluates the effectiveness of policies and practices aimed at fostering equitable career progression, and examines the inclusivity of their organizational culture.
2. UN Women and UN Global Compact. It establishes the Women’s Empowerment Principles (WEPs): This operational framework helps private companies promote gender equality and women’s empowerment within their organizations or community. Rather than measuring, it generates GLI criteria to influence public opinion internationally.
3. The IRIS+ System evaluates social and environmental factors, as well as gender equality. However, this evaluation system is aimed only at investment funds (not at individual companies or entrepreneurs) and therefore focuses on risks and returns in investment decisions.
4. The Small Assistance Fund (SEAF) Gender Equality Scorecard assesses six factors: pay equity, workforce participation, leadership in governance, benefits or professional development, workplace environment, and women-powered value chains. However, these measurements are aimed only at small and medium enterprises (SMEs).
5. The 2X Criteria was launched to help investors assess gender-smart businesses, targeting both funds and companies equally. They measure risk and impact on the one hand and gender aspects on the other. The criteria have been globally adopted and supported by IRIS+, HIPSO (Harmonized Indicators for Private Sector Operations), the Organization for Economic Cooperation and Development’s (OECD), UN Women’s WEPs. They have also been adopted by the International Finance Corporation (IFC).

To our knowledge, Small Enterprise Assistance Funds (SEAF) has generated the first tool to identify small businesses founded by women, through which diversity can be promoted via private funds. This identification is based on the six vectors aforementioned (2020). The inception of GLI focuses on female entrepreneurship because it stimulates aggregate demand, enhances the consumption of final goods and services, and contributes to economic and social development, thereby fostering more equitable societies by empowering women to secure their own livelihoods (Aljarodi et al., 2021).

The economic independence of women can pose significant challenges, especially for those who are single mothers or of foreign origin (Yin et al., 2023). Therefore, it is crucial to undertake the implementation of the commitments established in the 2030 Agenda, the most comprehensive global action plan worldwide, by all subscribing countries, to ensure the respect for the rights of women and girls. Although not the primary focus of this article, the support for female entrepreneurship is instrumental in integrating economically dependent family members into society, such as elderly relatives, as explored in the “Silver Economy” (Argentum, 2024) or dependent minors (Becker, 1991).

Following SEAF, the social enterprise Pro Mujer was founded in 1999 to help women develop their economic potential in Latin America. Together with Deetkeen and with the support of the United States Agency for International Development (USAID), they developed a document called “Tools for Investing with a Gender Perspective” (2021). These tools are divided into four sub-lenses that structure the topics of the yearly GLI Latam forums from 2020 to 2024, namely: (1) Women in leadership; (2) Equality in the workplace; (3) Products and services that benefit women and girls; (4) Equality in the value chain and advocacy practices. This document marked the initial comprehensive endeavor to measure gender equality within a company, alongside its gender-oriented investment strategy. “Tools for Investing with a Gender Perspective” assesses internal management policies and collaboration with civil society, as well as other local or global organizations. The document evaluates the efforts of organizations or funds to offer integrated services, including financial assistance, health, and educational loans, to low-income women in Latin America and the Caribbean.

A condensed version of this document, titled “Self-diagnosis of Gender Approach in Management,” has also been developed by Pro Mujer in partnership with Deekten Impact in their joint fund, the Ilu Women’s Empowerment Fund. This fund represents the first gender lens investing fund aimed at empowering women and advancing gender equality in Latin America and the Caribbean (Ilu Women's Empowerment Fund, 2021). Unlike the comprehensive assessment, this self-diagnosis tool focuses solely on evaluating a company’s management of gender lens investing rather than its investment actions in civil society. Its aim is to categorize a company as meeting the GLI criteria for receiving gender lens investments. The purpose of this exercise is to enable organizations to make informed decisions, prioritize actions, and effectively implement their commitment to gender equality. The results of the assessment outline three possible levels of advancement (basic, intermediate, or advanced) and recommend referring to the tools available for each lens.

Two years later, the PNC Financial Services Group, introduced six criteria to gender lens investors: gender diversity, women in leadership, women founders and fund managers, women-majority enterprise, gendered policies and practices, and products and services tailored to meet the needs of women (2023). “Diversity” appears to be the only aspect not explicitly addressed in the sub-lenses of the Ilu Women’s Empowerment Fund questionnaire, but it is a theme that emerges when discussing the second lens (“equality”).

EDGE auditing provides EDGE Certification, recognized as the leading global standard for Diversity, Equity, and Inclusion (DE&I) (EDGE Certified Foundation, 2024). The EDGE Standards and the Certification System are built on four pillars:

1. Representation at all levels of the organization, with particular emphasis on boards of directors and senior management.
2. Pay equity.
3. Effectiveness of policies and practices to ensure equitable career progression, including equal pay for equivalent work, recruitment and promotion processes, leadership development training and mentoring, flexible working arrangements, and organizational culture.
4. Inclusiveness of the culture, as indicated by employees’ ratings regarding career development opportunities (EDGE, 2024). Although the self-diagnosis developed by the Ilu Women’s Empowerment Fund in 2021 does not explicitly mention “culture,” it is implicitly considered within the metrics of “value chain and advocacy practice.” Like the Ilu Women’s Empowerment Fund, EDGE aims for equity at the workplace; specifically, a minimum of 30% female representation at all levels of companies. However, achieving this 30% threshold poses a greater challenge in boards of directors and senior management (reports from the national securities market commission).

Regarding the 2X Criteria, it can be utilized by any entity to establish their own benchmarks for new business and portfolios, as well as for self-reporting. Specifically, the 2X Criteria aids in identifying eligible investments through four criteria focused on the company itself: (1) entrepreneurship; (2) leadership; (3) employment; and (4) consumption of products and services for women (2X Challenge, 2024). Additionally, it incorporates an indirect criterion, “investments through financial intermediaries.” Therefore, it aligns with SEAF’s emphasis on small businesses and women entrepreneurs, as well as with the Ilu Women’s Empowerment Fund’s four sub-lenses, while also incorporating aspects such as “diversity” (PNC), “culture” (EDGE), and frameworks developed by the IRIS+ System and WEPS. Notably, the 2X Criteria is regarded as the global standard for gender finance.

Female participation and involvement in decision making processes is a component of a “virtuous circle” that brings economic and social benefits to new generations, particularly emphasizing young women (Daher et al., 2022). In Spain, an innovative program has been implemented to promote gender equality and bolster female leadership in the professional realm. Known as “Destino Talento” this initiative is the result of an agreement between *Closingap* and 50&50 GL, aimed at mentoring young women to achieve specific objectives (50&50 Gender Leadership, 2023). The primary objective of this program is to enhance women’s self-esteem and self-confidence, focusing on digital transformation and the values society needs, in line with the #ODS hashtags.

As more investors seek transparency regarding the criteria for selecting Gender Lens Investing (GLI) within portfolio companies, there is an increasing urgency to develop an integral tool for measuring and evaluating GLI policies and practices. Efforts to measure investment through a gender lens so far appear to complement one another; in fact, many of these initiatives have been supported by public funding from the UN (e.g., UN Women, UN Global Compact, and the 2X Criteria), which seem to advocate for an international tool that can assist their Supreme Audit Institutions (SAIs). However, no existing tool currently combines the strengths of the methods mentioned above. Therefore, the primary objective of this research is to propose a tool for self-assessing the impact of Gender Lens Investment (GLI) within companies, addressing the gaps left by previous measurement methods. Several organizations have already aimed to measure investment through a gender lens; however, to our knowledge, no academic metrics validated by both company and academic experts in Gender Lens Investing (GLI) have been identified.

Considering this, the purpose of this study is to develop a tool that will assist large national and international companies in measuring their organizational practices with a gender lens to establish a metric. This will facilitate their identification by investment fund consultants and inclusion in portfolios sought by investors with a gender lens. To accomplish this goal, a comprehensive review of prior tools, in collaboration with a board of experts, has provided critical insights into: (a) the dimensions to be incorporated in a metric designed to identify exemplary organizational practices in Gender Lens Investing (GLI); (b) the specific aspects of GLI organizational practices that merit particular emphasis; and (c) the prioritized importance of these identified dimensions based on expert evaluation.

As demonstrated, the objectives of the aforementioned organizations in measuring Gender Lens Investing (GLI) have been varied, encompassing legal compliance, support for women facing exclusion, the promotion of women-led small businesses, and enabling companies to self-assess their management practices and social investments in women-supportive activities. Consequently, firms should adopt proactive organizational mechanisms to empower and reinforce gender balance, considering the established benefits associated with this approach (Saeed et al., 2023).

Given these diverse dimensions, managers, mid-level professionals, and gender experts should combine efforts to develop a standardized metric applicable to both medium-sized and large enterprises. In this context, the Delphi method serves as a structured approach to address the current lack of consensus on such a metric.

After this introduction, the second section outlines the Delphi methodology, and the sequential phases employed to validate the questionnaire based on the identified dimensions, Part 3 describes the results and in section discussion and conclusions are presented.

## 2 Materials and methods

The Delphi is a widely used method in the context of research, especially in the field of Business and Social Sciences (Cabero Almenara and Infante Moro, 2014; Reguant-Alvarez and El Torrado-Fonseca, 2016). Many studies have shown its effectiveness to collect an expert view on different topics (Guldenmund, 2007; Fife-Schaw, 2020).

It is a valuable tool to assess the rigor and relevance of the items of a questionnaire based on the opinion of a group of experts through repeated consultations as they have several opportunities to give and revise their opinion (Giannarou and Zervas, 2014). It is an iterative process (López Gómez, 2018), controlled, guaranteeing the anonymity of the experts who receive statistical feedback of the overall response of the participants derived from the different rounds (Reguant-Alvarez and El Torrado-Fonseca, 2016) and allows consensus to be obtained avoiding direct confrontation between them, groupthink (Holeman et al., 2024) or that a few dominate the process (Boulkedid et al., 2011) and other types of influences or biases (Santaguida et al., 2018). Ultimately, it is characterized by anonymity, iteration and controlled feedback (Cuhls, 2023). These characteristics allow obtaining a balanced group response based on statistical data.

### 2.1 Delphi stages

In the context of the preparation and execution of a Delphi study, a series of steps are deployed that are often grouped into different stages. The number and names of these stages vary according to the literature consulted. Within the framework of this research (Bravo Estévez and Arrieta Gallastegui, 2005), three fundamental phases have been identified: preparatory, exploratory and final.

### 2.2 Preparatory phase

The following tasks were carried out during the first phase, preparatory:

- Configuring the Coordination Group: it is composed of the four authors who participated in the research.
- Literature review: an exhaustive search was conducted for papers related to ways of measuring the impacts of LIGs. The literature review made it possible to recognize the particularities of isolated recommendations on how to measure LIGs. For this reason, a set of items was included in the expert survey.
- Elaboration and revision of the GLI questionnaire for its validation. The coordinating team prepared the questionnaire, and, after its revision, 3 dimensions related to each of the GLI variables and 1 dimension with sociodemographic data were included (see Image 1). Each dimension included different objectives with a 6-point Likert scale, where 1 was totally disagree and 5 was totally disagree, 6 was do not know/no answer, open questions because they offer very valuable information (Hung et al., 2008, p. 197) and multiple response questions (See Table 1).

**Table 1:** Dimensions, related objectives and number of items of the questionnaire.

Dimension	Objective	Number of Likert-type scale items.	Number of items with open-ended questions
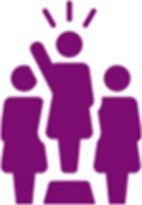 Leadership	Objective 1: To know if the company conducts diagnostics on women’s participation in the company	7	3
	Objective 2: To know if the company has a commitment to gender equality and gender mainstreaming in business management.	17	
	Objective 3: To know if the company has an inclusive governance approach with a gender perspective.	14	
	Objective 4: Find out if the company develops actions among the organization’s strategies to promote the participation and presence of women in decision-making and management positions.	5	
Equal employment opportunity 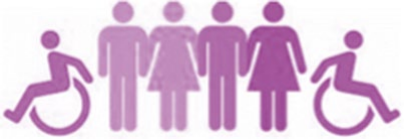	Objective 5: To determine whether the company has implemented work-life balance measures	11	1
	Objective 6: Find out whether the company takes equal pay into account.	4	
	Objective 7: Find out if the company has protocols in place to prevent and address workplace harassment (of any kind), violence, including sexual harassment	8	
	Objective 8: To determine whether the company measures the work environment from a gender perspective.	5	
Equality of products and services that the company offers to the Market 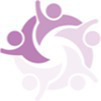	Objective 9: Find out if the company develops or provides any product or service with a gender perspective.	5	1

		Multiple choice questions	Number of items with open-ended questions
Sociodemographic 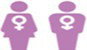	Gender, year of birth, educational levels, work experience, time worked, category of organization, sector, year of creation, size of company, job category, % of women in the company, % of women in management positions, % of women on board of directors or similar and others	19	3
Total		95	8

Own elaboration.

To evaluate the expertise of these two GLI experts, the Competence Coefficient (Kcomp) proposed by García-Martínez et al. (2012, p. 212) was applied, which is calculated as follows:


$$\mathrm{Kcomp}=0.5^{*}\;\left(Kc+Ka\right)$$

In this expression, Kc is obtained from the self-assessment made by the experts, and Ka is obtained from the professional experience and the number of publications (See Table 2). Both experts obtain scores above 0.8; therefore, they can be considered as valid because their level of competence is high.

**Table 2:** Criteria for the argumentation coefficient.

Sources of argumentation	Degrees of influence of sources on their knowledge and judgement		
Professional experience 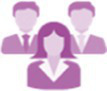	15 o more years	Between 5 and 10 years	5 years or less
	0.5	0,4	0,25
Publications related to the object of study 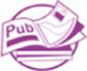	Scientific publications	Scientific publications	No Publication
	0,5	0,4	0,25
Total	1	0,8	0,5

Adapted from Paris, 2015.

The items of first draft of questionnaire were submitted for evaluation through the Delphi technique during the month of January 2024.

- Elaboration of the questionnaire for the first round of the Delphi:

The questionnaire sought the experts’ opinions on the clarity and relevance of the items related to each of the dimensions considered in GLI and their corresponding measurement scales (Table 1).

**Figure 1:**
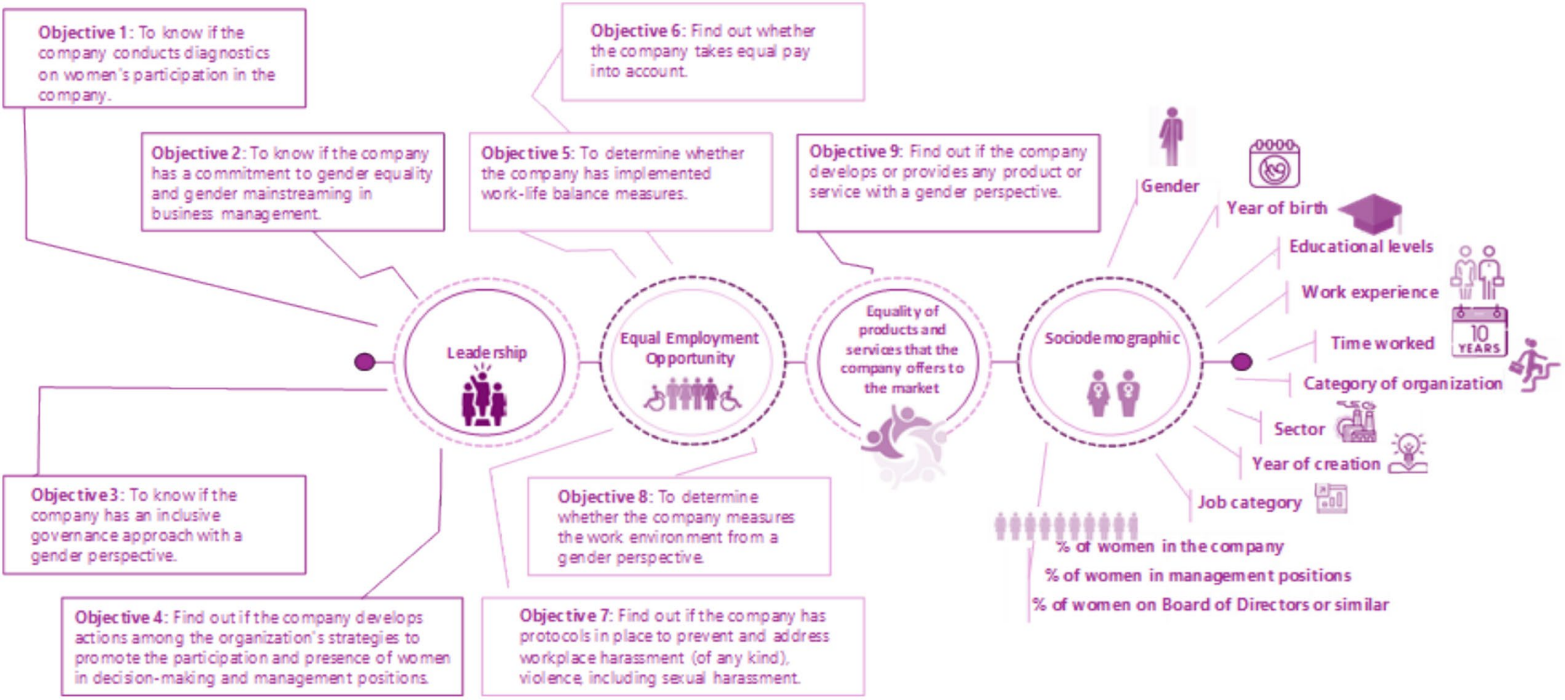
Dimensions and objectives related to GLIMetrics. Own elaboration.

Figure 1 presents the dimensions and objectives that, once considered most outstanding antecedents, our proposed questionnaire to measure practices centered on gender lens investing should include in any company.

- Selection of the panel of experts:

The selection of experts was one of the key aspects for the validity of the Delphi results. In this sense, the criteria for the selection of experts and the number of experts selected depended on the topic to be addressed and the objective to be achieved with the application of the Delphi method (appointments) and to avoid a high number of experts, since in this case the “dropout and rejection rate” is higher (Reid and Reid, 1988; Ortega Mohedano, 2008). According to Varela-Ruiz et al. (2012), this technique makes it possible to congregate knowledge increased by the concurrence of various experts.

Given the specificity of the object of study and the lack of previous studies, a small panel of experts was chosen. The coordinating team invited 20 potential experts who met the selection criteria. Ultimately, 10 of them agreed to participate in the study. The size of the panel is appropriate, given that, although there are clear discrepancies in the academic community as to the optimal number of panellists (minimum 5, maximum 30), it is within the parameters of representativeness (Zartha Sossa et al., 2019).

In this phase, the aim is to obtain a collective and diverse view of the experts on GLI through successive rounds of questions.

The objective criteria for the selection of the panellists (see Table 3) were as follows:

- Gender: Priority was given to the participation of women.
- Work Experience: Panellists with more than 15 years of experience were considered.
- Educational level: At least a bachelor’s degree was required and, preferably, higher education (master’s or doctorate).
- Age: Representatives of generations Z, Y, X and Baby Boomers were included.
- Professional category: Diversity was sought, from technical to managerial or middle management positions.
- Position: many panelists are employed in roles that entail diversity and gender responsibilities, or in human resources department.
- Professional recognition: through participation in scientific or professional publications, some of the panelists are members of relevant professional associations.
- Sector: Panellists were selected from organizations belonging to different economic activities.
- Preparation of the final questionnaire for the first round of the Delphi:

**Table 3:** Panel of experts who participated in the study.

Id	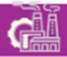 Sector	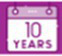 Time worked	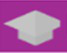 Educational levels	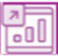 Job category	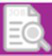 Position	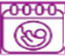 Year of birth
1	Manufacturing industry	+ 20 years	Master’s degree	Middle Management Position	Human Resources Management	1965–1980
2	Education and Training	+ 20 years	Doctorate	Executive Position	Vicepresident	1946–1964
3	Professional Services and Consulting	+ 20 years	Master’s degree	Executive Position	Principal Partner -Insurance Leader	1965–1980
4	Energy and Environment	+ 20 years	Bachelor’s Degree, University Degree, Engineering or similar	Technical Position (from all departments: HR, financial,…)	Performance Management	1965–1980
5	Others (Specify): Entertainment and leisure	+ 20 years	Master’s degree	Executive Position	Corporate Director of Talent Acquisition	1965–1980
6	Others (Specify): Media Agency	+ 20 years	Bachelor’s Degree, University Degree, Engineering or similar	Middle Management Position	Customer Service Director	1965–1980
7	Finance, banking and insurance	+ 20 years	Master’s degree	Middle Management Position	Deputy Director of International Affairs	1965–1980
8	Technologies/Computer Science and Telecommunications	16–20 years	Master’s degree	Middle Management Position	Talent Management Leader	1981–1996
9	Manufacturing industry	+ 20 years	Master’s degree	Middle Management Position	Development Manager	1946–1964
10	Professional Services and Consulting	+ 20 years	Master’s degree	Executive Position	Director of Experience and Culture	1965–1980

Own elaboration.

In this phase, two experts (a university professor and a manager of a multinational company in the financial sector, both with extensive knowledge and experience in gender) were asked to propose improvements to the questionnaire.

Their suggestions involved including some questions (Example: 6.4. Salaries are equal for men and women in identical job profiles; 7.11. In my company, people who take advantage of reconciliation measures have the same career development opportunities as the rest of the staff) and that we included the alternative Do not know/No answer.

The above suggestions were incorporated into the questionnaire that was finally sent to the panel of experts. Before sending the questionnaire, the coordinating team sent an email describing the objective, the process, a QR code and an electronic link to the Microsoft Forms questionnaire.

### 2.3 Exploratory phase

During this exploratory phase, two rounds of expert consultations were carried out to reach a consensus on the appropriateness and validity of the GLI items and its measurement scale.

- First round: the questionnaire proposed by the coordinating team was sent to the 10 experts so that they could give their opinion on the suitability of the items chosen for measuring the GLI. The questionnaire was divided into 10 blocks corresponding to the 3 dimensions of GLI. In each block, the expert is asked to indicate whether he/she considers that these questions correctly measure the aspects they are intended to measure. If he/she considers that the question is inadequate, he/she is asked to propose an alternative question and/or to make any suggestions or appreciations he/she may have in this regard. At the end of each section of the questionnaire, the expert was asked if, based on his or her professional experience, he or she could identify other aspects of GLI not covered by the study. This first round was carried out during week 3 of the month of January and February 2024.
- Second round: after processing the responses and analysing the overall results of the first round, the coordinating team prepared a report with the results obtained in the first round. Table 4 shows the level of consensus during the rounds.

**Table 4:** Level of expert consensus during the rounds.

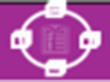 Level of consensus		
Agreement (A)	Neutral (N)	Disagreement (D)
Mdn ≥ 4 and IQR ≤ 1.5		Mdn ≤ 3.5 and IQR ≤ 1.5
or	If Mdn ≥ 3,5 and IQR ≤ 2	or
If Mdn ≥ 4 and IQR ≤ 2 and f (4–5) 70%		If Mdn ≤ 3.5 and IQR ≤ 2 and f (1–3) 70%

Mdn = median; IQR = interquartile range. Adapted from Mengual-Andrés et al. (2016).

After analyzing the comments and suggestions made by the experts, the questionnaire to be sent out in the second round was prepared, including information on the degree of agreement on each question and most of the suggestions revolving around the terminology used. This second round was carried out during the month of June 2024.

In this second round, the experts were asked to re-evaluate their responses considering the new information obtained in the first round in search of consensus.

### 2.4 Final phase

**Figure 2:**
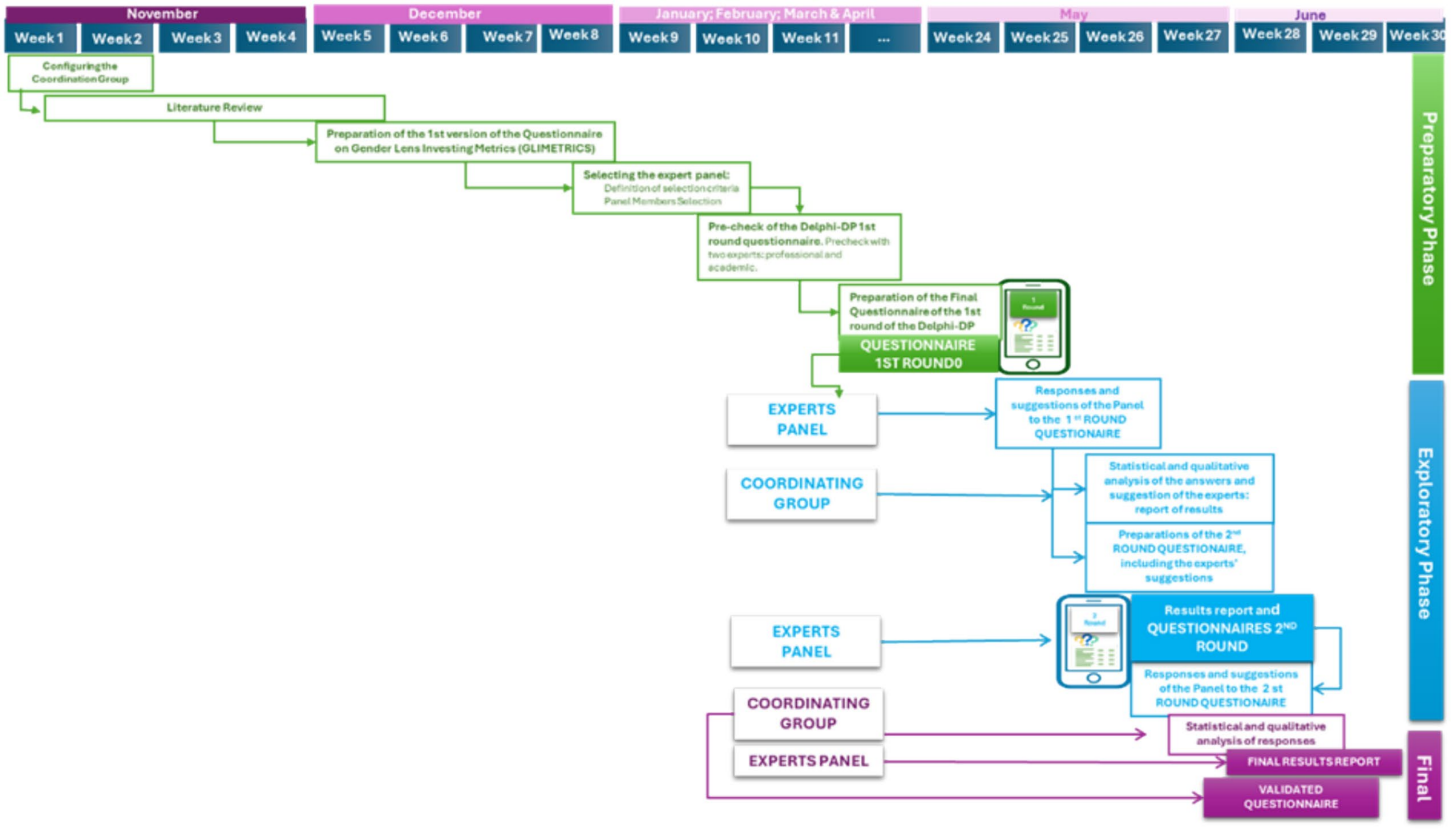
Steps followed in the 3 phases of the research. Own elaboration.

At the end of second round, a statistical analysis was carried out to quantify the responses, aggregate values and obtain a score that reflects the consensus among the experts. As a result of the whole process, the definitive and validated GLI items were generated and incorporated into the GLI metrics questionnaire. Figure 2 shows an outline of the steps followed in the 3 phases that have been carried out in this study.

## 3 Results

Once the information from the first round was collected, the responses of the 10 experts were analyzed on 76 items grouped into 11 objectives around each of the 3 dimensions identified, GLI and leadership, GLI and labor equality, and GLI on Equality of products and services offered by the company to the market, and on 19 items related to socio-demographic variables and gender. The experts responded on a Likert scale of 1 to 5, where 1 was strongly disagree, 2 was disagree, 3 was neither agree nor disagree, 4 was agree, and 5 was strongly agree.

Although there is no single way of determining when a consensus is reached among the different experts consulted in the Delphi, in this study it is understood to be reached under the criteria established by Mengual-Andrés et al. (2016) and López Gómez (2018). The median was chosen as the indicator of central tendency, supported by the interquartile range (IQR) (López Gómez, 2018; Margherita et al., 2021). Furthermore, the median value is very close to the mean, indicating that the distribution is approximately symmetrical.

Several studies have used 70% as the cutoff point (Lee et al., 2013; Campbell et al., 2018). Taking this into account, approximately 70% of GLI and Leadership were agreed, excluding the following items: 2.5, 2.10, 2.11, 2.12, 2.13, 2.14, 2.15, 2.16, 2.17, 3.2, 3.3, 3.4, 3.5, 3.6, 3.7, 3.8, 3.9, 3.10, 3.11, 4.1, 4.2, 4.3, 4.4, and 4.5. Tables 5–7 shows the results of the first round of the Delphi.

**Table 5:** Round I Delphi results: GLI and leadership.

Dimension 1: GLI and leadership						Quartile					
	Mean	Mo	Mdn	SD	CV	1	3	IQR	RIR	*f* (4-5)	CONS^1^
Objective 1: To know if the company conducts diagnostics on women's participation in the company											
1.1. The company identifies the gaps between women and men.	4.6	5	5	0.97	21%	5	5	0	0	90%	A
1.2. The company identifies areas where women are under-represented.	4.4	5	5	0.97	22%	4	5	1	0.2	90%	A
1.3. The company identifies: the number of employees promoted.	4.3	5	5	1.49	35%	5	5	0	0	80%	A
1.4. The company identifies: the number of female board members.	4.1	5	5	1.73	42%	4.25	5	0.75	0.15	80%	A
1.5. The company identifies: the number of female managers.	4.6	5	5	0.97	21%	5	5	0	0.15	90%	A
1.6. The company analyses the barriers to women's access to decision-making positions.	3.9	5	4.5	1.45	37%	3.25	5	1.75	0.39	70%	A
1.7. The results of the above analyses are used to design strategies to promote the participation of women in decision-making and management positions.	4	5	4	1.05	26%	3.25	5	1.75	0.44	80%	A
Objective 2: To know whether the organisation is committed to gender equality and gender mainstreaming in management.											
2.1. My organization's mission statement includes a commitment to gender equality.	4.3	5	5	1.06	0.25	4	5	1	0.2	*80%*	A
2.2. Gender equality is included in my organization's objectives.	4.4	5	4.5	0.7	0.16	4	5	1	0.22	*90%*	A
2.3. My company formally acknowledges its responsibility for equality to its workforce and to society	4.6	5	5	0.7	0.15	4.25	5	0.75	0.15	*90%*	A
2.4. An equality plan is in place.	4.6	5	5	0.84	0.18	5	5	0.00	0	*80%*	A
2.6. The equality plan is published on the website to give effect to the organization's commitment.	4.22	5	5	1.09	0.26	4	5	1	0.2	*70%*	A
2.7. My organization has specific gender awareness and training programs for all staff.	4.1	5	4	0.99	0.24	4	5	1	0.25	*80%*	A
2.8. Employees confirm that their views are considered in the organization's internal actions on gender issues.	3.9	5	4	1.2	0.31	3.25	5	1.75	0.44	*70%*	A
2.9. HR implements strategies and concrete actions to neutralize possible gender bias in selection processes.	4.3	5	4.5	0.82	0.19	4	5	1	0.22	*80%*	A
Objective 3: To know if the company has an inclusive governance approach with a gender perspective.											
3.1. There is a strategic document setting out the organization's internal actions.	3.9	4	4	0.99	0.25	3.25	4.75	1.5	0.38	*70%*	A
3.12. The strategic plan has been approved by senior management.	4.22	5	5	1.2	0.28	3	5	2	0.4	*67%*	A
3.13. Senior management is committed to the plan	4.11	5	5	1.17	0.28	3	5	2	0.4	*67%*	A

1 Mdn = median; Mean = arithmetic mean; SD = standard deviation; CV = coefficient of variation; IQR = interquartile range; RIR = relative interquartile range; *f* (4–5) = frequency of assessment 4 and 5; CONS = level of consensus. Own elaboration.

**Table 6:** Round I Delphi results: GLI and equal employment opportunity.

Dimension 2: GLI and equal employment opportunity						Quartile					
	Mean	Mo	Mdn	SD	CV	1	3	IQR	RIR	*f* (4–5)	CONS^1^
Objective 5: To determine whether the company has implemented work-life balance measures											
5.1. The company has implemented some work-life balance measures (for example: teleworking, flexible schedules, compressed work weeks, paid and unpaid leave, workroom...).	4	5	5	0.53	0.13	4	5	1	0.2	100%	A
5.2. Reconciliation measures are published.	3.89	4	4	0.53	0.14	4	5	1	0.25	100%	A
5.3. There is a known process for requesting conciliation measures.	4	5	5	0.53	0.13	4	5	1	0.2	100%	A
5.4. Reconciliation measures avoid stereotypes and roles.	3.67	5	4	0.83	0.23	4	5	1	0.25	78%	A
5.9. In my company, the need to reconcile work and family life is seen as something that concerns both male and female employees.	3.78	4	4	0.71	0.19	4	5	1	0.25	89%	A
5.11. In my company, people who use conciliation have the same career opportunities as the rest of the staff.	3.89	4	4	1.05	0.27	3	5	0.5	0.5	67%	A
Objective 7: Find out if the company has protocols in place to prevent and address workplace harassment (of any kind), violence, including sexual harassment.											
7.1. The company publicly supports zero tolerance.	4	5	5	0.76	0.19	4	5	1	0.2	88%	A
7.2. A protocol is in place to prevent and address workplace violence, including all forms of harassment	4.56	5	5	1.01	0.22	5	5	0	0	89%	A
7.3. The protocol is made public.	4.56	5	5	1.01	0.22	5	5	0	0	89%	A
7.4. The mechanisms for activating the protocol are clear.	4.33	5	5	1.12	0.26	4	5	1	0.2	78%	A
7.5. The mechanisms for activating the protocol are friendly	4.22	5	5	1.09	0.26	4	5	1	0.2	78%	A
7.6. The Protocol guarantees confidentiality.	4.56	5	5	1.01	0.22	5	5	0	0	89%	A
7.8. The people who design and implement the protocol are trained in gender equality, workplace violence, sexual harassment and bullying.	4.5	5	5	1.27	0.28	4	5	1	0.2	67%	A
Objective 8: To determine whether the company measures the work environment from a gender perspective.											
8.1. A work climate survey is conducted with a gender perspective.	3.9	5	4	1.2	0.31	3.25	5	1.75	0.44	70%	A
8.2. It is applied to all personnel on a regular and confidential basis.	4.1	5	4.5	1.1	0.27	3.25	5	1.75	0.39	70%	A
8.5. The persons responsible for the design and implementation of the protocol receive training in the following areas: gender equality, workplace violence, sexual harassment and bullying.	4.13	5	4.5	1.13	0.27	3.75	4.75	1.25	0.28	75%	A

1 Mdn = median; Mean = arithmetic mean; SD = standard deviation; CV = coefficient of variation; IQR = interquartile range; RIR = relative interquartile range; f (4–5) = frequency of assessment 4 and 5; CONS = level of consensus. Own elaboration.

**Table 7:** Round I Delphi results: GLI and equality of products and services that the company offers to the market.

Dimension 3: GLI and equality of products and services that the company offers to the market						Quartile					
	Mean	Mo	Mdn	SD	CV	1	3	IQR	RIR	*f* (4–5)	CONS^1^
Objective 9: Find out if the company develops or provides any product or service with a gender perspective.											
9.3. Information from different genders (customers) is utilised in an equal manner for the purpose of product or service development.	3.89	4	4	1.05	0.27	3	5	2	0.5	67%	A
9.6. In customer service, care is taken not to reproduce gender stereotypes.	4.5	5	5	0.97	0.22	4.25	5	0.75	0.15	90%	A

1 Mdn = median; Mean = arithmetic mean; SD = standard deviation; CV = coefficient of variation; IQR = interquartile range; RIR = relative interquartile range; *f* (4–5) = frequency of assessment 4 and 5; CONS = level of consensus. Own elaboration.

As it can be observed in Tables 5–7, the results demonstrate a high level of consensus among participants with regard to the identification of gender gaps, the implementation of measures designed to promote equality and reconciliation, and the existence of anti-harassment protocols. The median score for these items on the first Delphi round questionnaire is 4 or above, indicating agreement. This value remains constant in the second and third quartiles, indicating that the majority of responses are concentrated in Likert scale scores 4 or 5. This confirms a relatively symmetrical distribution. The interquartile range (IQR) reaches a maximum value of 2 (≤ 1.5), indicating a low dispersion of responses. Moreover, the proportion of ratings 4 and 5 (agree, strongly agree) is at least 70% (≥ 70%), reaching 100% in some cases. Except for items 3.12, 3.13, 5.11 and 7.8, which were included with 67% of ratings 4 and 5. The remaining items were included because they met the other conditions and were close to the 70% level of agreement.

The data presented above indicates that a consensus has been reached among the experts participating in the initial round of the Delphi study with respect to the appropriateness and clarity of the GLI items illustrated in Tables 5–7. For items related to GLI Leadership, and GLI Quality of products and services that the company offers to the market, the frequency of ratings 4 and 5 on the Likert scale is 79%, and for GLI Equal Employment Opportunity 83%. Applying the acceptance criteria, we observe that, for all items, the median ≥ 4 and IQR ≤ 1.5 are met. Subsequent to the aforementioned conclusions, the coordinating group proceeded to conduct a second round of questioning, encompassing those items which were identified as exhibiting discrepancies of opinion and failing to satisfy the requisite conditions:

As illustrated in Table 4, the medians (4 and 5) indicate a predominantly positive perception of the assessed areas, while the interquartile ranges (IQRs) vary, demonstrating a divergence in consensus among respondents. The data demonstrate a slight positive skewness. This is characterized by a mean that is lower than both the median and mode. However, there are instances where the mean, median and mode are almost equivalent. This suggests that the distribution is more symmetrical. In round II, all experts who participated in the first round participated too. The results of the second round are shown in Tables 8–10.

**Table 8:** Results of Delphi round II GLI and leadership.

Dimension 1: GLI and leadership						Quartile					
	Mean	Mo	Mdn	SD	CV	1	3	IQR	RIR	*f* (4–5)	CONS^1^
Objective 2: To know whether the organization is committed to gender equality and gender mainstreaming in management.											
2.5. The staff is regularly informed about the actions of the equality plan.	3.80	4.00	4.00	1.03	0.27	4.00	4.00	0.00	0.00	80%	A
2.10. Training programs are in place to promote women to management positions.	3.50	2.00	3.50	1.27	0.36	2.50	4.75	2.50	0.71	50%	N
2.11. Staff are periodically asked for their opinion on internal actions on gender issues.	3.30	4.00	3.50	1.34	0.41	2.50	4.00	1.75	0.50	50%	N
2.12. Employees are regularly asked for their opinion on internal actions on gender issues.	2.60	2.00	2.50	1.07	0.41	2.00	3.00	1.00	0.40	10%	D
2.13. Gender differences are regularly reviewed in my organization.	3.30	3.00	3.00	1.06	0.32	3.00	4.00	1.00	0.33	40%	N
2.14. More men than women are employed on temporary contracts.	2.30	3.00	2.50	1.06	0.46	1.25	3.00	1.75	0.70	10%	D
2.15. Permanent contracts are more common for men than for women.	2.20	3.00	2.50	0.92	0.42	1.25	3.00	1.75	0.70	0%	D
2.16. Is there a job/occupation where only men have been recruited?	3.10	4.00	3.50	1.37	0.44	2.25	4.00	1.75	0.50	50%	N
2.17. Is there any job/occupation where only women have been recruited?	2.90	4.00	3.00	1.37	0.47	2.00	4.00	2.00	0.67	40%	N
Objective 3: To know if the company has an inclusive governance approach with a gender perspective.											
3.2. There is a strategic document that states the organization’s actions at the external level.	3.70	4.00	3.85	0.95	1.04	3.00	4.00	1.00	0.26	60%	N
3.3. The document includes guidelines for the design of equality policies in the four investment lenses with a gender perspective: Women in leadership.	3.30	3.00	3.15	0.95	0.95	3.00	4.00	1.00	0.32	40%	N
3.3. The document contains guidelines for the design of gender equality policies in the four investment lenses: Women in leadership.	3.30	4.00	3.50	0.82	0.25	3.00	4.00	1.00	0.29	50%	D
3.5. The document provides guidelines for the design of equality policies in the four investment lenses from a gender perspective: –Products and services that benefit women.	3.00	3.00	3.00	0.82	0.27	2.25	3.75	1.50	0.50	30%	D
3.6. The document provides guidelines for designing gender equality policies in the four investment lenses from a gender perspective: –Girls and gender in the value chain and advocacy practices.	2.70	3.00	3.00	0.67	0.25	2.00	3.00	1.00	0.33	10%	D
3.7. The strategic plan includes detailed objectives, targets and indicators by gender.	3.50	3.00	3.50	1.08	0.31	3.00	4.00	1.00	0.29	50%	N
3.8. The strategic plan includes concrete actions to be taken.	3.70	4.00	4.00	0.95	0.26	3.00	4.00	1.00	0.25	60%	N
3.9. The strategic plan identifies those responsible for its implementation (names and surnames).	3.10	2.00	3.00	1.10	0.36	2.00	4.00	2.00	0.67	40%	N
3.10. The strategic plan includes deadlines.	3.60	4.00	4.00	1.07	0.30	2.00	4.00	2.00	0.50	60%	N
3.11. There is a budget for the implementation of the plan.	3.30	2.00	3.50	1.25	0.38	3.00	4.00	1.00	0.29	50%	N
3.14. In my organization there is sensitivity toward Trans and non-Binary groups.	3.90	4.00	4.00	1.20	0.31	2.00	4.00	2.00	0.50	80%	A
Objective 4: To find out if the company develops measures within the organisation’s strategies to promote the participation and presence of women in decision-making and management positions.											
4.1. A parity ratio of 50% is established for the presence of women in decision-making and management positions.	2.80	3.00	3.00	1.14	0.41	2.00	3.00	1.00	0.33	20%	D
4.2. Special measures are activated for promotion (e.g., women candidates on all pre-selection lists, or favoring the promotion of women in the case of exclusive positions for women).	3.33	2.00	2.00	1.06	0.46	2.00	2.75	0.75	0.33	20%	D
4.3. There is a gender balance in all areas of your organization.	3.83	2.00	2.00	0.82	0.36	2.00	2.75	0.75	1.33	10%	D
4.4. There is a gender balance in work teams in your organization.	1.17	3.00	2.00	1.03	0.47	1.25	3.00	1.75	1.33	10%	D
4.5. In your organization there is gender balance in ownership.	0.33	1.00	2.00	1.16	0.50	3.25	4.00	0.75	1.33	20%	D

1 Mdn = median; Mean = arithmetic mean; SD = standard deviation; CV = coefficient of variation; IQR = interquartile range; RIR = relative interquartile range; *f* (4–5) = frequency of assessment 4 and 5; CONS = level of consensus. Own elaboration.

**Table 9:** Results of Delphi round II GLI and equal employment opportunity.

Dimension 2: GLI and equal employment opportunity						Quartile					
	Mean	Mo	Mdn	SD	CV	1	3	IQR	RIR	*f* (4–5)	CONS^1^
Objective 5: To determine whether the company has implemented work-life balance measures											
5.6. The company analyses the results of work-life balance from a gender perspective.	3.90	4.00	4.00	0.74	0.18	3.25	4.00	0.75	0.18	70%	A
5.7. Working hours are always respected with or without the use of work-life balance measures.	3.40	4.00	3.50	0.96	0.28	3.00	4.00	1.00	0.28	50%	N
5.8. Maternity and paternity leave is extended beyond the legal deadlines.	2.80	3.00	3.00	1.13	0.40	2.00	3.00	1.00	0.33	202%	D
5.10. In my company, the percentage of (male) fathers using work-life balance measures is higher than the average of other companies.	2.90	2.00	3.00	0.94	0.34	2.00	3.00	1.00	0.33	20%	D
Objective 6: To find out whether the company takes equal pay into account.											
6.1. Salary scales shall be published solely on the basis of the objective characteristics of each post.	3.10	1.00	3.50	1.72	0.55	1.25	4.75	3.50	1.00	50%	N
6.2. Salaries and salary increases are determined through a documented and transparent process.	2.70	3.00	3.00	1.16	0.42	1.75	4.75	3.00	1.00	30%	D
6.3. Publish data disaggregated by gender and by level of position in terms of remuneration.	2.80	3.00	3.00	1.31	0.47	2.00	3.75	1.75	0.58	30%	D
6.4. Salaries are equal for men and women in identical job profiles.	3.60	4.00	4.00	1.35	0.37	2.00	3.75	1.75	0.43	60%	N
Objective 7: Find out if the company has protocols in place to prevent and address workplace harassment (of any kind), violence, including sexual harassment.											
7.7. The protocol takes into account events outside the physical workplace (e.g., virtual or other environments).	3.80	4.00	4.00	1.03	0.27	3.00	4.75	1.75	0.44	60%	N
Objective 8: To determine whether the company measures the work environment from a gender perspective.											
8.3. Based on the diagnosis, identify different gender gaps and opinions.	4.00	4.00	3.60	1.08	0.29	3.00	4.00	1.00	30.00	30.0%	D
8.4. The results are used to address the problems identified and to guide the action plan to implement measures to promote gender equality in the workplace.	4.00	4.00	3.70	0.95	0.26	3.00	4.00	1.00	30.00	20.0%	D

1 Mdn = median; Mean = arithmetic mean; SD = standard deviation; CV = coefficient of variation; IQR = interquartile range; RIR = relative interquartile range; *f* (4–5) = frequency of assessment 4 and 5; CONS = level of consensus. Own elaboration.

**Table 10:** Results of Delphi round II GLI and equal employment opportunity.

Dimension 3: GLI and equality of products and services that the company offers to the market						Quartile					
	Mean	Mo	Mdn	SD	CV	1	3	IQR	RIR	*f* (4-5)	CONS^1^
Objective 9: Find out if the company develops or provides any product or service with a gender perspective.											
9.1. Participation of all genders is ensured in all work teams involved in the process of a product or service.	3.30	3.00	3.00	0.82	0.25	3.00	3.75	0.75	0.25	30.00	D
9.2. Strategies are used to understand gender needs, interests and expectations.	2.80	3.00	3.00	0.63	0.23	2.50	3.00	0.75	0.25	10.00	D
9.4. We consider the context and product- or service-related inequalities experienced by customers on the basis of gender.	2.90	3.00	3.00	0.32	0.11	3.00	3.00	0.00	0.00	0.00	D
9.5. Reducing gender inequalities through the product or service is considered a bottom line priority.	2.30	2.00	2.00	0.67	0.29	2.00	3.00	1.00	0.50	0.00	D

1 Mdn = median; Mean = arithmetic mean; SD = standard deviation; CV = coefficient of variation; IQR = interquartile range; RIR = relative interquartile range; *f* (4–5) = frequency of assessment 4 and 5; CONS = level of consensus. Own elaboration.

As shown in Table 9, the values of all statistical parameters improve with respect to the previous round, indicating that the redrafting of the questions following the suggestions of the experts has strengthened the degree of consensus among them. For items related to GLI and Leadership, the frequency of ratings 4 and 5 on the Likert scale is 38%; GLI and Equal Employment Opportunity 57% and for GLI and Equality of Products and Services that the company offers to the Market 10%. Applying the acceptance criteria, we observe that for all items, the median ≥ 4 and IQR ≤ 1.5, are met.

In Table 11, both rounds are compared to find out the stability of the panel, which is understood as the consistency in the experts’ opinions between successive rounds of the Delphi, regardless of their degree of convergence (Pozo et al., 2007). In Table 8, the different parameters analyzed in both rounds are compared, and improvements are shown.

**Table 11:** Comparison of results in Rounds I and II.

					Quartile					
GLI	Rounds	Mdn	Mean	SD	1	2	3	IQR	*f* (4–5)	CONS^1^
GLI1	R1	4.00	3.73	0.67	3.75	4.00	4.00	0.25	80%	A
	R2	4.50	4.50	0.53	4.00	4.50	5.00	1.00	100%	A
GLI2	R1	4.00	4.09	0.57	3.00	4.00	4.00	1.00	90%	A
	R2	4.00	4.27	0.67	4.00	4.00	5.00	1.00	90%	A
GLI3	R1	4.00	4.27	0.48	4.00	4.00	4.00	0.00	100%	A
	R2	4.50	4.50	0.53	4.00	4.50	5.00	1.00	100%	A
GLI4	R1	4.00	4.09	0.57	3.00	4.00	4.00	1.00	90%	A
	R2	5.00	4.64	0.52	4.00	5.00	5.00	1.00	100%	A

^1^Mdn = median; Mean = arithmetic mean; SD = standard deviation; CV = coefficient of variation; IQR = interquartile range; RIR = relative interquartile range; *f* (4–5) = frequency of assessment 4 and 5; CONS = level of consensus. Own elaboration.

**Figure 3:**
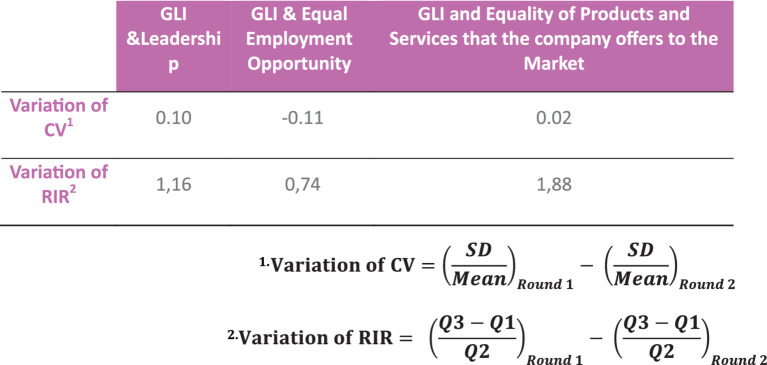
Variation of RIR and CV between rounds.

Stability occurs if the variation of the interquartile range between rounds is less than 0.30 and consensus is reached if the variation of the coefficient of variation between rounds is less than 0.40 (Mengual-Andrés et al., 2016) as shown in Figure 3.

Based on the results obtained, the Delphi is closed after the second round, given that the criteria for closing the Delphi are met, as there is a high degree of consensus (median and interquartile range) and great stability in the opinions of the experts between rounds (variation in RIR and CV between rounds).

A comparative analysis of the two rounds revealed notable variation in the coefficient of variation (CV) and relative interquartile range (RIR) across three key dimensions. The dimensions of GLI and Leadership, GLI and Equal Employment Opportunity, and GLI and Equality of Products and Services were examined.

With regard to GLI and Leadership, the CV demonstrated an increase of 0.10 units, thereby indicating a greater dispersion in the second-round data in comparison to the first round. Concurrently, the RIR increased by 1.16 units, indicating a greater degree of relative variability in the distribution of the data. In the domain of equal employment opportunities, the coefficient of variation (CV) decreased by 0.11 units, indicating a reduction in the dispersion of the data. Conversely, the relative interquartile range (RIR) increased by 0.74 units, suggesting greater relative variability. Lastly, with regard to equality in products and services offered by the company, the coefficient of variation (CV) exhibited a modest increase of 0.02 units, while the relative interquartile range (RIR) demonstrated a pronounced surge of 1.88 units, indicating a considerable variability in the distribution between the two rounds.

## 4 Discussion and conclusion

The primary objective of this study was to develop and validate a comprehensive questionnaire designed to identify and assess the presence and frequency of Gender Lens Investing (GLI) practices within firms, alongside providing recommendations for enhancement. Particular emphasis was placed on three key dimensions of GLI practices as highlighted in the literature and recent influential reports: GLI and leadership, GLI and labor equality, and GLI concerning the equity of products and services offered by the company to the market.

In this regard, it is surprising that Chmiel et al. (2017), when assessing personnel selection and assessment (PSA) processes and the effectiveness of Human Resource Management (HRM) in training, overlook the fact that gender bias acts as a significant ‘barrier to entry’ for certain jobs. Additionally, they fail to address the need for promoting equity within organizations, ensuring equal job opportunities for all. For Chmiel et al. (2017), the gender lens appears more as a problem than a necessity, which stands in contrast to the United Nations’ emphasis on ‘Gender Equality’ in SDG 5. More recent research by Pignault and Houssemand (2021) acknowledges that their study does not consider crucial social, cultural, or individual factors and fails to address gender issues. This omission suggests that occupational psychology has yet to fully integrate gender considerations into its research.

The fact that Sustainable Finance Geneva suggests five infrastructures with their respective measurement methods highlights the lack of consensus on these measurements. Considering the differences between these and later tools: (1) EDGE does not provide a numerical approach but rather an approximate one, and it neglects the needs of female consumers; (2) Sustainable Finance Geneva generates criteria rather than measuring, making it not a tool in itself but rather a set of recommendations for measurement; it explains how to create the tool but does not implement it; (3) The IRIS+ System supports investment fund managers but does not assist companies; (4) SEAF is limited to measuring small and medium-sized enterprises; (5) The 2XCriteria is more focused on measuring economic return than on internal gender policies within companies; and finally, (6) Pro-Mujer, in partnership with Deekten, concentrates on gender policies applicable to any company—whether local, national, or international—but, like the others, it lacks numerical measurement scales, offering only general assessments that would not be useful for evaluating investment funds.

Questionnaire validation prior to its deployment is crucial, as it ensures the quality, reliability, and validity of the data collected, as well as the appropriateness and comprehensibility of the questions for the target population. The Delphi method, widely recognized in both business and social science research, is especially valuable in contexts where information is implicit or biased. In this study, two rounds of expert consultation following the Delphi method were conducted to refine and validate the GLI metrics questionnaire. The statistical analysis of these rounds demonstrated high levels of consensus, stability, and agreement among experts. Following the first round, which showed a substantial level of agreement, experts contributed open-ended feedback and suggestions for improvement. This input allowed for the reformulation of certain items in the second round, incorporating expert insights on specific aspects of GLI organizational practices that warranted particular attention. The validated questionnaire in this study is based on the most developed tool created by the 2XCriteria, incorporating three of its key thresholds: leadership, equality, and products or services for women consumers. Additionally, it draws on key criteria from other relevant literature and aims to serve as an international model tool that consolidates these efforts. The questionnaire is intended to be a reference for Supreme Audit Institutions (SAIs). By providing numerical results that measure management practices and policies across various types of companies, the tool can be easily utilized by both national and international audits for local, national, or global companies within investment fund portfolios.

Through the Delphi stages, the study established the dimensions to be prioritized within the metric and refined the clarity and relevance of the items. Furthermore, the terminology used was adapted to align with language commonly employed by Spanish firms. The validated questionnaire developed through this research offers a new methodological tool with several practical implications: (1) identifying and measuring gender lens investing practices; (2) enabling self-assessment based on the established metric to guide organizational routines; (3) informing decision-making related to gender lens investing; and (4) allowing for the testing of propositions regarding the reciprocal influence between GLI practices and organizational outcomes. In short, this tool is designed to provide evidence of how gender lens organizational practices impact firm performance.

Additionally, the tool offers significant potential for raising awareness. It enables organizations and professional groups to recognize the importance of regularly investing in GLI as part of established organizational practices, which is essential for effectively measuring GLI impact. Furthermore, it provides researchers with a valuable new instrument to support comparative studies, facilitate generalization of case study findings, and explore potential correlations across diverse GLI approaches.

### 4.1 Limitations

The questionnaire developed in this study has yet to be implemented across multiple companies and, therefore, remains untested in practical organizational settings. This limited application is attributed to the instrument’s novelty, the necessity for further validation, and limited dissemination among organizations that could potentially benefit from its use. Regarding organizational practices, existing literature frequently emphasizes the outcomes of gender lens investments, such as increased female representation in leadership roles or enhanced pay equity indicators. However, there is comparatively less focus on the specific practices companies adopt to realize these outcomes.

### 4.2 Further research

Adopting a comprehensive approach that accounts for both organizational practices and the outcomes of gender equality investments will be essential. Further, a more rigorous academic analysis of these practices can yield valuable insights into the mechanisms that actively foster gender equality and inclusive leadership.

In conclusion, widespread implementation of the questionnaire across a diverse range of companies, along with a refined academic focus on organizational practices, is critical to advancing the understanding and promotion of workplace gender equality. Broadening the application of the questionnaire will not only allow for its validation but also enable refinement and enhancement of its components, ensuring its relevance across various sectors and organizational cultures. This approach supports comparative analysis and facilitates the potential generalization of findings derived from case studies.

## Data Availability

The raw data supporting the conclusions of this article will be made available by the authors, without undue reservation.
