# Supplementary material for: Developing an index for measuring gender lens investing in organizations: the GLIMETRICS framework
Source: Front Psychol. 2025 Apr 30;16:1534355. doi: 10.3389/fpsyg.2025.1534355 (PMC12075249; doi:10.3389/fpsyg.2025.1534355)
Supplement: Supplementary file 1 [file Data_Sheet_1.pdf]

DIMENSION 1: GLI AND LEADERSHIP
Objective 1: To know if the company conducts diagnostics on women's participation in the company
1.1. The company identifies the gaps between women and men.
1.2. The company identifies areas where women are under-represented.
1.3. The company identifies: the number of employees promoted.
1.4. The company identifies: the number of female board members.
1.5. The company identifies: the number of female managers.
1.6. The company analyses the barriers to women's access to decision-making positions.
1.7. The results of the above analyses are used to design strategies to promote the participation of women in decision-making and management positions.
Objective 2: To know whether the organisation is committed to gender equality and gender mainstreaming in management.
2.1. My organization's mission statement includes a commitment to gender equality.
2.2. Gender equality is included in my organization's objectives.
2.3. My company formally acknowledges its responsibility for equality to its workforce and to society
2.4. An equality plan is in place.
2.5. The staff is regularly informed about the actions of the equality plan.
2.6. The equality plan is published on the website to give effect to the organisation's commitment.
2.7. My organization has specific gender awareness and training programmes for all staff.
2.8. Employees confirm that their views are considered in the organisation's internal actions on gender issues.
2.9. HR implements strategies and concrete actions to neutralize possible gender bias in selection processes.
2.10. Training programmes are in place to promote women to management positions.
2.11. Staff are periodically asked for their opinion on internal actions on gender issues.
2.12. Employees are regularly asked for their opinion on internal actions on gender issues.
2.13. Gender differences are regularly reviewed in my organisation.
2.14. More men than women are employed on temporary contracts.
2.15. Permanent contracts are more common for men than for women.
2.16. Is there a job/occupation where only men have been recruited?
2.17. Is there any job/occupation where only women have been recruited?
Objective 3: To know if the company has an inclusive governance approach with a gender perspective.
3.1. There is a strategic document setting out the organisation's internal actions.
3.2. There is a strategic document that states the organization's actions at the external level.
3.3. The document includes guidelines for the design of equality policies in the four investment lenses with a gender perspective: Women in leadership.
3.4. The document includes guidelines for the design of equality policies in the four lenses of gender-sensitive investment: - Equality in the workplace.
3.5. The document provides guidelines for the design of equality policies in the four investment lenses from a gender perspective: - Products and services that benefit women.
3.6. The document provides guidelines for designing gender equality policies in the four investment lenses from a gender perspective: - Girls and gender in the value chain and advocacy practices.
3.7. The strategic plan includes detailed objectives, targets and indicators by gender.
3.8. The strategic plan includes concrete actions to be taken.
3.9. The strategic plan identifies those responsible for its implementation (names and surnames).
3.10. The strategic plan includes deadlines.
3.11. There is a budget for the implementation of the plan.
3.12. The strategic plan has been approved by senior management.
3.13. Senior management is committed to the plan
3.14. In my organization there is sensitivity towards Trans and non-Binary groups.
Objective 4: To find out if the company develops measures within the organisation's strategies to promote the participation and presence of women in decision-making and management positions.
4.1. A parity ratio of 50% is established for the presence of women in decision-making and management positions.
4.2. Special measures are activated for promotion (e.g. women candidates on all pre-selection lists, or favouring the promotion of women in the case of exclusive positions for women).
4.3. There is a gender balance in all areas of your organisation.
4.4. There is a gender balance in work teams in your organisation.
4.5. In your organisation there is gender balance in ownership.

DIMENSION 2: GLI AND EQUAL EMPLOYMENT OPPORTUNITY
Objective 5: To determine whether the company has implemented work-life balance measures
5.1. The company has implemented some work-life balance measures (for example: teleworking, flexible schedules, compressed work weeks, paid and unpaid leave, workroom...).
5.2. Reconciliation measures are published.
5.3. There is a known process for requesting conciliation measures.
5.4. Reconciliation measures avoid stereotypes and roles.
5.5. The company controls who and when a reconciliation measure is triggered.
5.6. The company analyses the results of work-life balance from a gender perspective.
5.7. Working hours are always respected with or without the use of work-life balance measures.
5.8. Maternity and paternity leave is extended beyond the legal deadlines.
5.9. In my company, the need to reconcile work and family life is seen as something that concerns both male and female employees.
5.10. In my company, the percentage of (male) fathers using work-life balance measures is higher than the average of other companies.
5.11. In my company, people who use conciliation have the same career opportunities as the rest of the staff.
Objective 6: Find out whether the company takes equal pay into account.
6.1. The salary scales are published based solely on the objective characteristics of each position.
6.2. Remunerations, as well as increases, are established in a documented and transparent process.
6.3. Data disaggregated by gender and level of positions according to remuneration is published.
6.4. Wages are equal for men and women in identical job profiles.
Objective 7: Find out if the company has protocols in place to prevent and address workplace harassment (of any kind), violence, including sexual harassment.
7.1. The company publicly supports zero tolerance.
7.2. A protocol is in place to prevent and address workplace violence, including all forms of harassment
7.3. The protocol is made public.
7.4. The mechanisms for activating the protocol are clear.
7.5. The mechanisms for activating the protocol are friendly
7.6. The Protocol guarantees confidentiality.
7.7. The protocol takes into account events outside the physical workplace (e.g. virtual or other environments).
7.8. The people who design and implement the protocol are trained in gender equality, workplace violence, sexual harassment and bullying.
Objective 8: To determine whether the company measures the work environment from a gender perspective.
8.1. A work climate survey is conducted with a gender perspective.
8.2. It is applied to all personnel on a regular and confidential basis.
8.3. Based on the diagnosis, identify different gender gaps and opinions.
8.4. The results are used to address the problems identified and to guide the action plan to implement measures to promote gender equality in the workplace.
8.5. The persons responsible for the design and implementation of the protocol receive training in the following areas: gender equality, workplace violence, sexual harassment and bullying.

DIMENSION 3: GLI AND EQUALITY OF PRODUCTS AND SERVICES THAT THE COMPANY OFFERS TO THE MARKET
9.1. Participation of all genders is ensured in all work teams involved in the process of a product or service.
9.2. Strategies are used to understand gender needs, interests and expectations.
9.3. Information from different genders (customers) is utilised in an equal manner for the purpose of product or service development.
9.4. We consider the context and product- or service-related inequalities experienced by customers on the basis of gender.
9.5. Reducing gender inequalities through the product or service is considered a bottom line priority.
9.6. In customer service, care is taken not to reproduce gender stereotypes.
